# Aldosterone Impairs Mitochondrial Function in Human Cardiac Fibroblasts via A-Kinase Anchor Protein 12

**DOI:** 10.1038/s41598-018-25068-6

**Published:** 2018-05-01

**Authors:** Jaime Ibarrola, Rafael Sadaba, Ernesto Martinez-Martinez, Amaia Garcia-Peña, Vanessa Arrieta, Virginia Alvarez, Amaya Fernández-Celis, Alicia Gainza, Victoria Cachofeiro, Enrique Santamaria, Joaquin Fernandez-Irigoyen, Frederic Jaisser, Natalia Lopez-Andres

**Affiliations:** 1 Cardiovascular Translational Research, Navarrabiomed, Complejo Hospitalario de Navarra (CHN), Universidad Pública de Navarra (UPNA), IdiSNA, Pamplona, Spain; 2 Department of Physiology, School of Medicine, Universidad Complutense, Instituto de Investigación Sanitaria Gregorio Marañón (IiSGM), Ciber de Enfermedades Cardiovasculares (CIBERCV), Instituto de Salud Carlos III, Madrid, Spain; 3 Proteored-ISCIII, Proteomics Unit, Navarrabiomed, Complejo Hospitalario de Navarra (CHN), Universidad Pública de Navarra (UPNA), IdiSNA, Pamplona, Spain; 4 INSERM, Centre d’Investigations Cliniques-Plurithématique 1433, UMR 1116 Université de Lorraine, CHRU de Nancy, French-Clinical Research Infrastructure Network (F-CRIN) INI-CRCT, Nancy, France; 5 INSERM UMRS 1138 Team 1, Centre de Recherche des Cordeliers, University Pierre and Marie Curie, Paris, France

## Abstract

Aldosterone (Aldo) contributes to mitochondrial dysfunction and cardiac oxidative stress. Using a proteomic approach, A-kinase anchor protein (AKAP)-12 has been identified as a down-regulated protein by Aldo in human cardiac fibroblasts. We aim to characterize whether AKAP-12 down-regulation could be a deleterious mechanism which induces mitochondrial dysfunction and oxidative stress in cardiac cells. Aldo down-regulated AKAP-12 via its mineralocorticoid receptor, increased oxidative stress and induced mitochondrial dysfunction characterized by decreased mitochondrial-DNA and Peroxisome proliferator-activated receptor gamma coactivator 1-alpha (PGC-1α) expressions in human cardiac fibroblasts. CRISPR/Cas9-mediated knock-down of AKAP-12 produced similar deleterious effects in human cardiac fibroblasts. CRISPR/Cas9-mediated activation of AKAP-12 blunted Aldo effects on mitochondrial dysfunction and oxidative stress in human cardiac fibroblasts. In Aldo-salt-treated rats, cardiac AKAP-12, mitochondrial-DNA and PGC-1α expressions were decreased and paralleled increased oxidative stress. In myocardial biopsies from patients with aortic stenosis (AS, n = 26), AKAP-12, mitochondrial-DNA and PGC-1α expressions were decreased as compared to Controls (n = 13). Circulating Aldo levels inversely correlated with cardiac AKAP-12. PGC-1α positively associated with AKAP-12 and with mitochondrial-DNA. Aldo decreased AKAP-12 expression, impairing mitochondrial biogenesis and increasing cardiac oxidative stress. AKAP-12 down-regulation triggered by Aldo may represent an important event in the development of mitochondrial dysfunction and cardiac oxidative stress.

## Introduction

Mitochondria are complex intracellular organelles involved in energy production, reactive oxygen species (ROS) generation and regulation of cell death pathways^1^. Functional and structural alterations of mitochondria promote an increase in ROS production^2,3^. Mitochondrial DNA (mtDNA), which encodes essential protein components of the mitochondrial oxidative phosphorylation complexes, is prone to oxidative stress^4^. Moreover, defects in biogenesis results in reduced number of mitochondria^5^, leading to increased ROS generation and detrimental consequences on cardiac function^6^. Mitochondrial biogenesis is controlled by peroxisome proliferator-activated receptor gamma coactivator 1-alpha (PGC-1α), which could regulate mtDNA replication^7^.

Aldosterone (Aldo), a mineralocorticoid hormone primarily synthesized in the adrenal gland, is a major regulator of extracellular fluid volume and sodium and potassium balance^8^. Numerous studies since over two past decades have shown that Aldo plays a role in the development of different cardiovascular diseases^9–11^ and the pathophysiological basis has been related to its ability to induce oxidative stress^11^. Chronic exposure to ROS leads to cardiac apoptosis, fibrosis and dysfunction^12^. It has been demonstrated that mitochondrial dysfunction mediates Aldo-induced podocyte damage as well as epithelial-mesenchymal transition in renal proximal tubular epithelial cells^13,14^.

A kinase anchoring proteins (AKAPs), compose a growing list of diverse but functionally related proteins defined by their ability to bind to the regulatory subunit of protein kinase A^15^. The roles of AKAPs are to localize, specify, amplify, and accelerate intracellular signal transduction by linking upstream signal generators to downstream effectors or by recruiting multiple signaling enzymes within signaling hub^16^. AKAPs family has been shown to participate in the pathogenesis of cardiac arrhythmia, heart failure and hypertrophy^16^. Moreover, some AKAPs have an important role in the modulation of ROS synthesis. AKAP-121 (also known as AKAP-1) down-regulation contributes to the development of cardiac dysfunction by increasing ROS levels and promoting cell death in cardiac cells^17^. AKAP-12 is also known to cause significant down-regulation of hypoxia inducible factor 1-α and thereby reduce the hypoxia induced by vascular endothelial growth factor expression^18^.

Our group recently identified that Aldo down-regulated AKAP-12 expression in human cardiac fibroblasts using a proteomic approach^19^. Therefore, the present study was designed to analyze whether AKAP-12 down-regulation could be a deleterious mechanism by which Aldo induces mitochondrial dysfunction and oxidative stress in cardiac cells, in myocardium from Aldo-salt-treated rats and in myocardial biopsies from aortic stenosis (AS) patients.

## Results

### Aldo decreased AKAP-12 and altered mitochondrial function and oxidative stress in adult human cardiac fibroblasts

**Figure 1:**
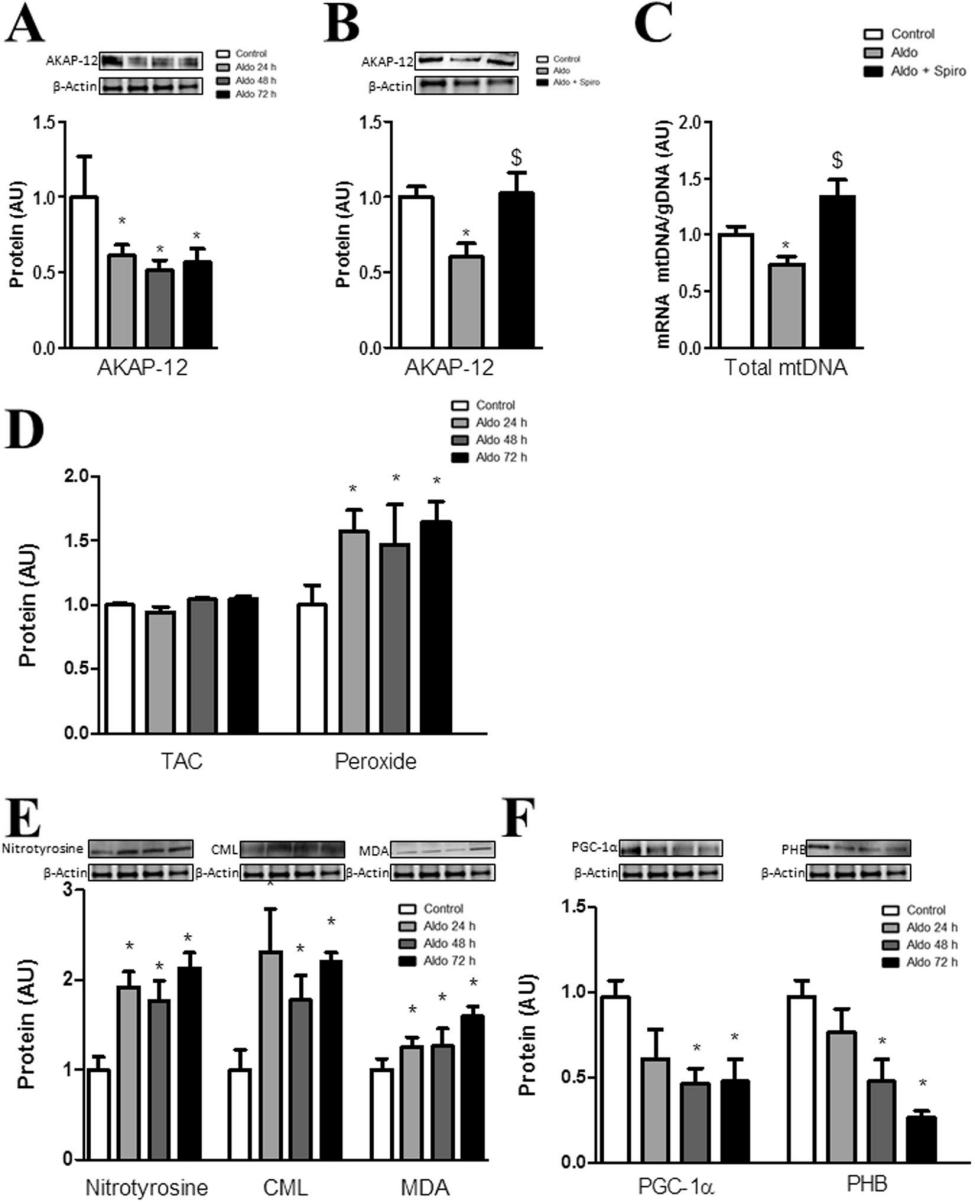
Aldo modulates AKAP-12 and regulates oxidative stress in adult human cardiac fibroblasts. Aldo effects on AKAP-12 protein expression in human cardiac fibroblasts (**A,B**). Total mtDNA expression was measured in human cardiac fibroblasts (**C**). Oxidative markers (**D,E**) and mitochondrial function markers (**F**) expressed as a fold change relative to controls in lysate cells from Aldo-treated human cardiac fibroblasts. All conditions were performed at least in triplicate. Histogram bars represent the mean ± SEM of 6 assays. For Western blot experiments, the blots were cropped, protein densitometry was expressed in arbitrary units (AU) once normalized to β-actin. *p < 0.05 vs Control. AKAP-12, A-kinase anchoring protein 12; Aldo, Aldosterone; Spiro, Spironolactone; Total mtDNA, Total mitochondrial DNA; TAC, Total antioxidant capacity; CML, carboxy-methyl-lysine; MDA, malondialdehyde; PGC-1α, peroxisome proliferator-activated receptor-gamma coactivator 1 alpha; PHB, prohibitin.

We have recently identified AKAP-12 as a down-regulated protein in Aldo-treated human cardiac fibroblasts using a global proteomic approach^19^. We analyzed time course of Aldo effect on AKAP-12 expression and showed that Aldo decreased (p < 0.05) the expression of AKAP-12 at 24, 48 and 72 hours (Fig. 1A). This down-regulation occurred via the mineralocorticoid receptor since Spironolactone (Spiro), a Mineralocorticoid Receptor Antagonist, blunted the Aldo effect (Fig. 1B).

Aldo also decreased (p < 0.05) total mtDNA and this effect was blocked (p < 0.05) by Spiro treatment (Fig. 1C). Total antioxidant capacity (TAC) production was not affected by Aldo treatment whereas peroxide production was increased in human cardiac fibroblasts (p < 0.05) (Fig. 1D). Nitrotyrosine and carboxy-methyl-lysin (CML) expressions were increased (p < 0.05) by Aldo at all the times analyzed while malondialdehyde (MDA) levels were enhanced (p < 0.05) only at 72 hours of treatment (Fig. 1E). PGC-1α and prohibitin (PHB) expressions were significantly reduced (p < 0.05) by Aldo treatment at 48 and 72 hours (Fig. 1F).

### AKAP-12 knock-down altered mitochondrial function and oxidative stress parameters in adult human cardiac fibroblasts

**Figure 2:**
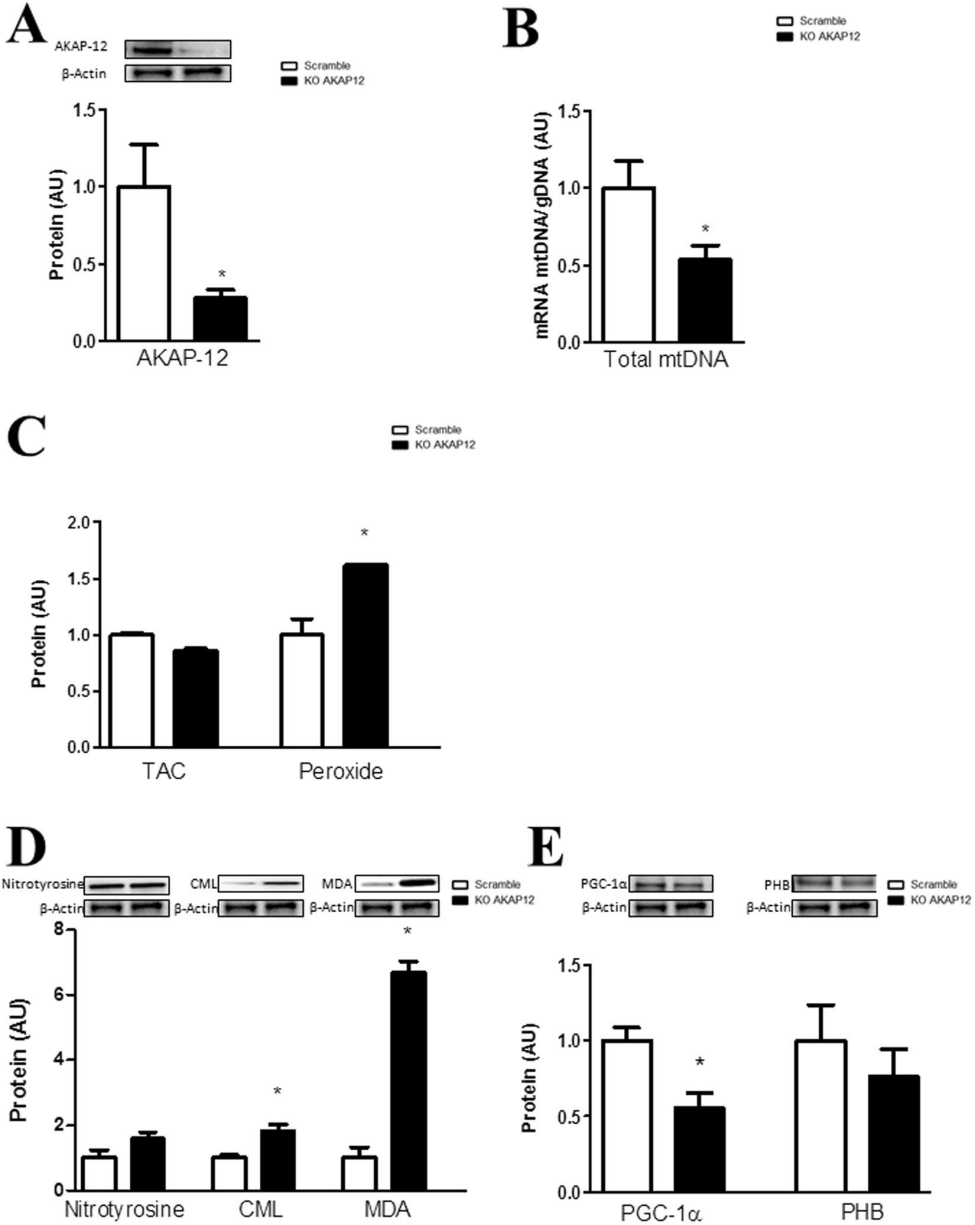
AKAP-12 inhibition modifies mitochondrial function and oxidant status in adult human cardiac fibroblasts. AKAP-12 protein (**A**) expression was measured in AKAP-12-knock-down human cardiac fibroblast. Total mtDNA expression was quantified by RT-PCR (**B**). Effects of AKAP-12 knock-down on oxidative markers (**C,D**). Effects of AKAP-12 knock-down on PGC-1α and PHB protein expressions (**E**). Histogram bars represent the mean ± SEM of 6 assays. For Western blot experiments, the blots were cropped, protein densitometry was expressed in arbitrary units (AU) once normalized to β-actin. For RT-PCR experiments, data was normalized to HPRT and β-actin for cDNA. *p < 0.05 vs Scramble. AKAP-12, A-kinase anchoring protein 12; Total mtDNA, Total mitochondrial DNA; TAC, Total antioxidant capacity; CML, carboxy-methyl-lysine; MDA, malondialdehyde; PGC-1α, peroxisome proliferator-activated receptor-gamma coactivator 1 alpha; PHB, prohibitin.

AKAP-12 knock-down cells presented reduced AKAP-12 protein levels (60%; p < 0.05) (Fig. 2A). Total mtDNA was decreased (p < 0.05) in AKAP-12 knock-down cells (Fig. 2B) as compared to Scramble. AKAP-12 depleted cells exhibited similar TAC levels whereas peroxide production was significantly increased (p < 0.05) in AKAP-12-silenced cells as compared to Scramble (Fig. 2C). AKAP-12 knock-down cells exhibited similar levels of nitrotyrosine, while CML and MDA expressions were significantly increased (p < 0.05) in AKAP-12-silenced cells as compared to Scramble (Fig. 2D). PGC-1α expression was reduced (p < 0.05) in AKAP-12-silenced cells as compared to Scramble, whereas prohibitin levels were not modified (Fig. 2E).

### AKAP-12 activation prevented Aldo effects on mitochondrial function and oxidative stress parameters in adult human cardiac fibroblasts

**Figure 3:**
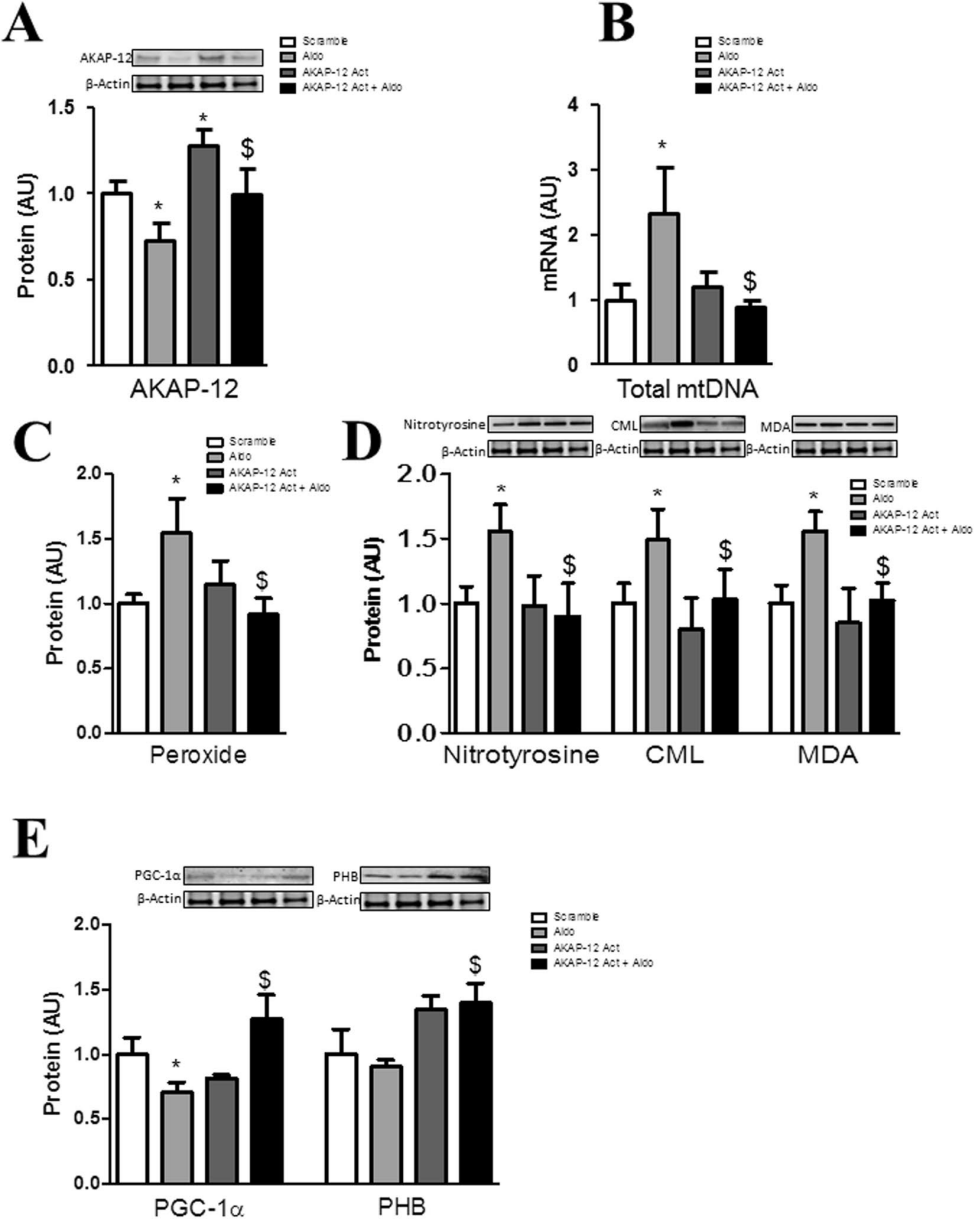
AKAP-12 activation prevented Aldo effects on mitochondrial function and oxidative stress parameters in adult human cardiac fibroblasts. AKAP-12 protein (**A**) expression was measured in human cardiac fibroblasts over-expressing AKAP-12. Total mtDNA expression was quantified by RT-PCR (**B**). Effects of AKAP-12 over-expression on peroxide production (**C**) and oxidative stress markers (**D**). Effects of AKAP-12 activation on PGC-1α and PHB protein expressions (**E**). Histogram bars represent the mean ± SEM of 6 assays. For Western blot experiments the blots were cropped, protein densitometry was expressed in arbitrary units (AU) once normalized to β-actin. For RT-PCR experiments, data was normalized to HPRT and β-actin for cDNA. *p < 0.05 vs Scramble. ^$^p < 0.05 vs Aldo. AKAP-12, A-kinase anchoring protein 12; Total mtDNA, Total mitochondrial DNA; TAC, Total antioxidant capacity; CML, carboxy-methyl-lysine; MDA, malondialdehyde; PGC-1α, peroxisome proliferator-activated receptor-gamma coactivator 1 alpha; PHB, prohibitin.

Cells transfected with AKAP-12 clustered regularly interspaced short palindrome repeats (CRISPR)/Cas9 activation plasmid presented increased AKAP-12 protein (35%, p < 0.05) (Fig. 3A) as compared to Scramble cells. The increase in total mtDNA induced by Aldo was restored (p < 0.05) in cells over-expressing AKAP-12 (Fig. 3B). Aldo-enhanced peroxide production was normalized (p < 0.05) in AKAP-12-activated cells (Fig. 3C). The effect of Aldo on nitrotyrosine, CML and MDA expressions (Fig. 3D) as well as PGC-1α or PHB expressions (Fig. 3E) was blunted in cells that overexpress AKAP-12.

### AKAP-12 expression, mitochondrial function and oxidative stress parameters in hearts from Aldo-salt-treated rats

**Figure 4:**
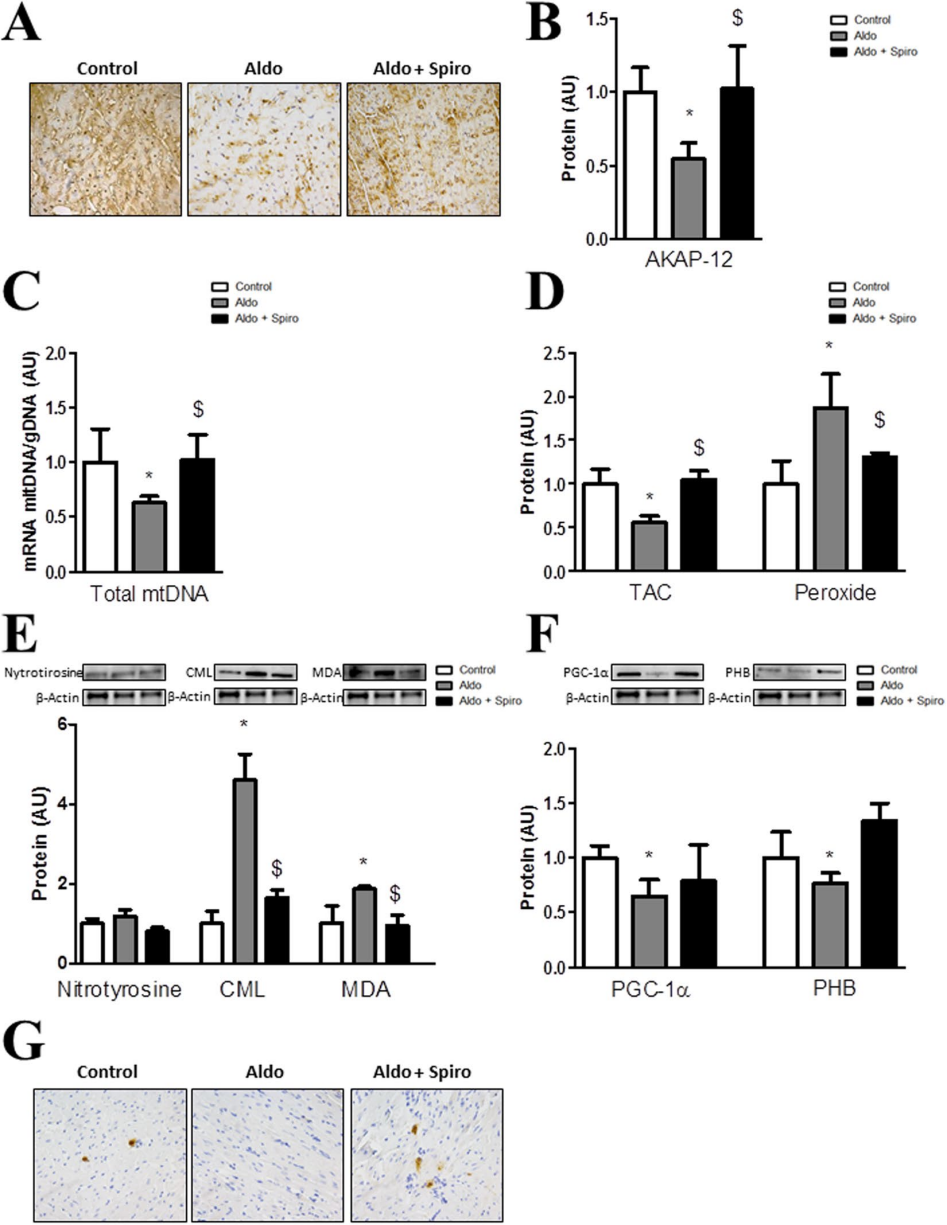
AKAP-12, mitochondrial function and oxidative stress markers in hearts from Aldosterone-salt-treated rats. AKAP-12 immunostaining and protein expression (**A,B**) in myocardium from Controls, Aldo-salt-treated rats and Aldo-salt + Spiro-treated rats. Total mtDNA expression was measured (**C**). TAC and peroxide production (**D**). Oxidative stress markers (**E**). PGC-1α and PHB expressions (**F**). PGC-1α immunostaining (**G**). Magnification of the microphotographs 40x. Histogram bars represent the mean ± SEM of each group of subjects. For Western blot experiments, the blots were cropped, protein densitometry was expressed in arbitrary units (AU) once normalized to β-actin. *p < 0.05 vs Control; ^$^p < 0.05 vs Aldo. AKAP-12, A-kinase anchoring protein 12; Aldo, Aldosterone; Spiro, Spironolactone; Total mtDNA, Total mitochondrial DNA; TAC, Total antioxidant capacity; CML, carboxy-methyl-lysine; MDA, malondialdehyde; PGC-1α, peroxisome proliferator-activated receptor-gamma coactivator 1 alpha; PHB, prohibitin.

AKAP-12 expression was reduced (p < 0.05) in myocardium from Aldo-salt-treated rats as compared to controls (Fig. 4B). Total mtDNA was significantly decreased (p < 0.05) in myocardium from Aldo-salt-treated rats as compared to controls (Fig. 4C). In hearts from Aldo-salt-treated animals, TAC was decreased (p < 0.05) and peroxide production was enhanced (Fig. 4D). Nitrotyrosine expression was not modified by Aldo-salt treatment whereas CML and MDA expressions were significantly increased (p < 0.05) as compared to controls (Fig. 4E). PGC-1α and PHB expressions were reduced (p < 0.05) in Aldo-salt-treated myocardium as compared to controls (Fig. 4F). Representative images of PGC-1α immunochemistry showed lower staining in hearts from Aldo-salt-treated rats (Fig. 4G). Treatment with Spiro normalized all the parameters analyzed (Fig. 4A–G).

### AKAP-12 expression, mitochondrial function and oxidative stress parameters in myocardial biopsies from patients with aortic stenosis

The baseline characteristics of the patients and controls are presented in Table 1. In keeping with the typical characteristics of patients presenting with AS, a significant proportion suffered from hypertension and were predominantly NYHA class II–III.

**Table 1:** Baseline characteristics of aortic stenosis patients and controls.

	Controls	AS patients
n	13	26
Age (years)	75 ± 11	74 ± 8
Male	7 (54%)	12 (46%)
Hypertension	1 (8%)	18 (69%)
Hyperlipidaemia	3 (23%)	16 (62%)
Diabetes	1 (8%)	2 (7%)
Coronary artery disease	1 (8%)	7 (27%)
Lung disease	4 (30%)	
Cause of death:		
-Bronchopneumonia	4 (30%)	
-Sepsis	1 (8%)	
-Cancer	6 (46%)	
-Trauma	1 (8%)	
-Old age	1 (8%)	
LVEF (%)		65 ± 13
NYHA		I (11.5%)
		II (57.7%)
		III (23.1%)
		IV (7.7%)
Aldosterone (pg/ml)		59.13 ± 32.7

Values are mean ± SD. LVEF = left ventricular ejection fraction; NYHA = New York Heart Association classification of heart failure; Aldo = Aldosterone.

**Figure 5:**
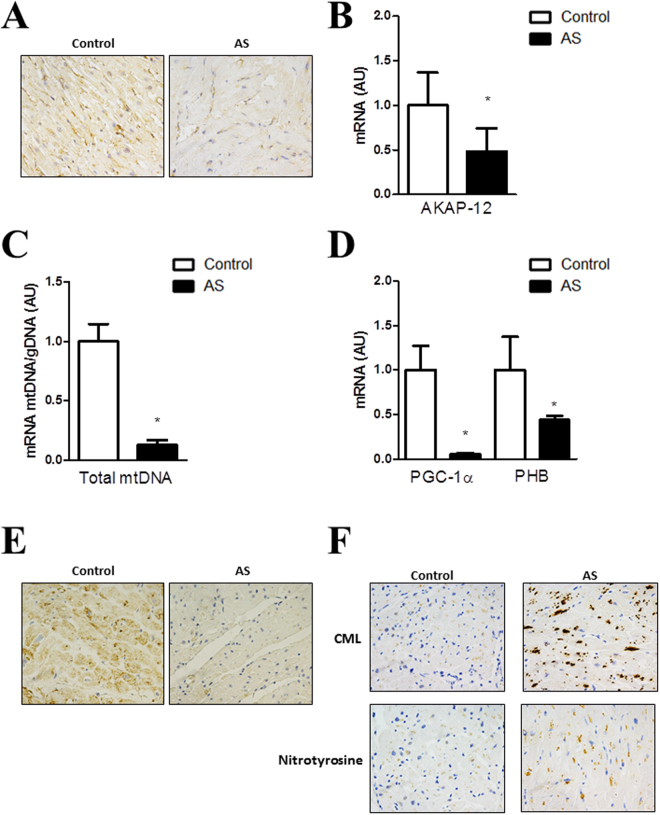
AKAP-12, mitochondrial function and oxidative stress markers in myocardial biopsies from aortic stenosis patients. AKAP-12 immunostaining and AKAP-12 mRNA levels in myocardial biopsies from AS patients (**A,B**). Total mtDNA expression was measured (**C**). PGC-1α and PHB mRNA levels (**D**) and PGC-1α immunostaining (**E**) in myocardial biopsies. Immunostaining of Nitrotyrosine and CML (**E**). Magnification of the microphotographs 40x. Histogram bars represent the mean ± SEM of each group of subjects (Control n = 13 and patients with AS, n = 26) in arbitrary units (AU) normalized to HPRT and β-actin for cDNA. *p < 0.05 vs. control group. AS, aortic stenosis; AKAP-12, A-kinase anchoring protein 12; Total mtDNA, Total mitochondrial DNA; PGC-1α, peroxisome proliferator-activated receptor-gamma coactivator 1 alpha; PHB, prohibitin; CML, carboxy-methyl-lysine.

AKAP-12 expression quantified by immunohistochemistry and RT-PCR was lower in myocardium from AS patients as compared to controls (Fig. 5A,B). Moreover, mtDNA was decreased (p < 0.05) in myocardium from AS patients as compared to controls (Fig. 5C). PGC-1α and PHB mRNA expressions were reduced (p < 0.05) in myocardium from AS patients (Fig. 5D). Accordingly, PGC-1α immunostaining was lower in hearts from AS patients (Fig. 5E). AS myocardial biopsies presented increased nitrotyrosine and CML immunostainings (Fig. 5F).

### Association studies in patients with aortic stenosis

**Figure 6:**
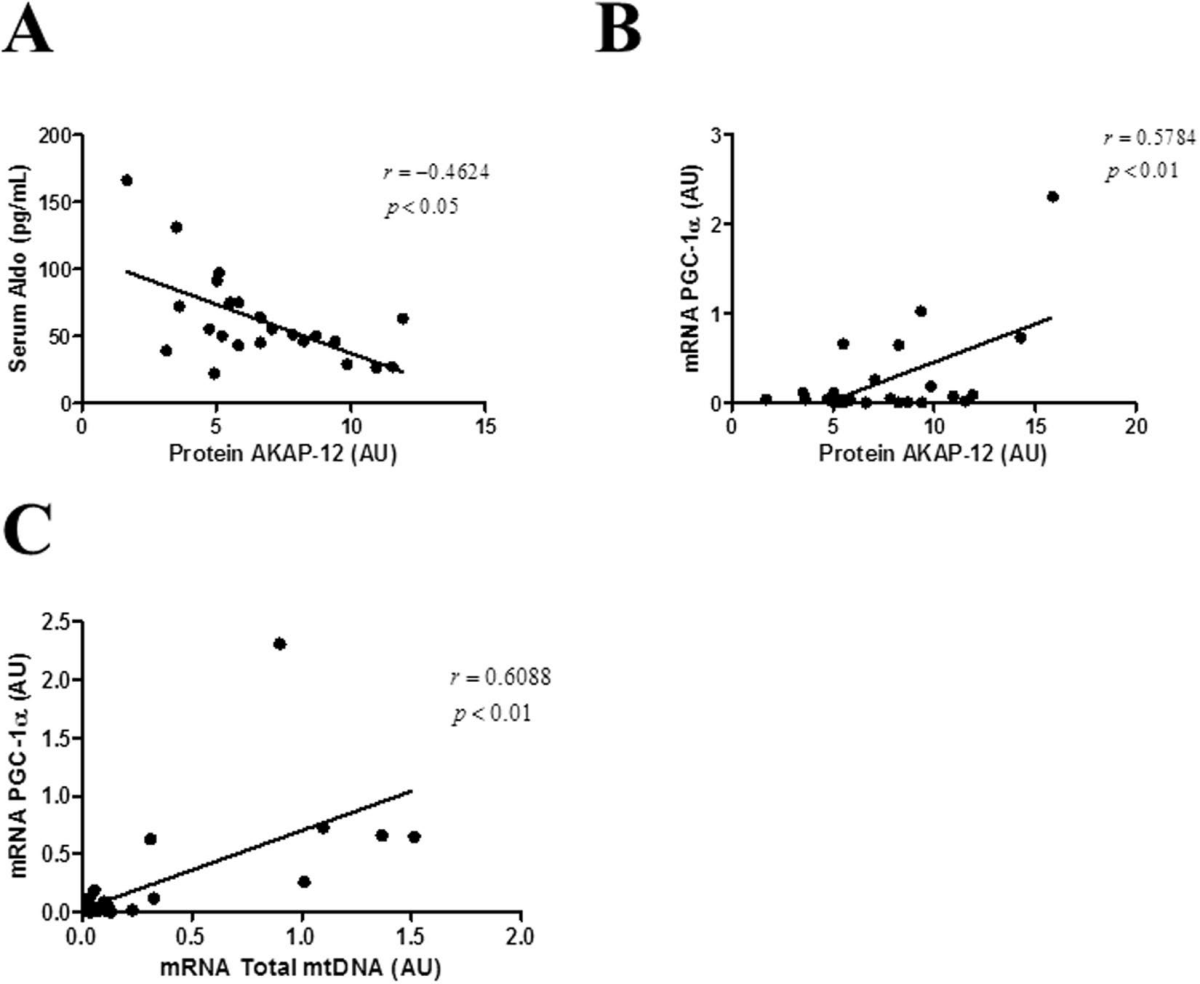
Correlation studies in aortic stenosis patients. AKAP-12 protein expression negatively correlated with serum Aldo (**A**) and positively correlated with PGC-1α mRNA levels (**B**) in AS patients. Total mtDNA levels positively correlated with PGC-1α mRNA levels in the whole population (**C**). AKAP-12, A-kinase anchoring protein 12; Aldo, Aldosterone; PGC-1α, peroxisome proliferator-activated receptor-gamma coactivator 1 alpha.

AKAP-12 protein levels inversely correlated with serum Aldo (r = −0.4624, p < 0.05; Fig. 6A) in AS patients and positively correlated with PGC-1α mRNA levels (r = 0.5784, p < 0.01; Fig. 6B) in the whole population. Interestingly, PGC-1α mRNA levels positively correlated with Total mtDNA (r = 0.6088, p < 0.01 Fig. 6C) in all patients.

## Discussion

Using a quantitative proteomic approach, we found that AKAP-12 was differentially down-regulated in Aldo-treated human cardiac fibroblasts as compared to controls. Aldo treatment as well as AKAP-12 down-regulation induced mitochondrial dysfunction and oxidative stress markers in human cardiac fibroblasts. This effect was mediated by mineralocorticoid receptor activation. Interestingly, over-expression of AKAP-12 in human cardiac fibroblasts blunted Aldo-induced mitochondrial dysfunction and oxidative stress. In parallel to AKAP-12 down-regulation, mitochondrial dysfunction and oxidative stress markers were enhanced in myocardium from Aldo-salt-treated rats and blunted by mineralocorticoid receptor antagonist. In AS patients, circulating Aldo levels negatively correlated to myocardial AKAP-12 expression. In myocardial biopsies from AS patients, AKAP-12 down-regulation paralleled and associated to mitochondrial dysfunction.

Some AKAPs proteins have been previously related to mitochondrial function, oxidative stress and in the development of the hypertrophic response induced by Aldo in cardiomyocytes^20^. Indeed, AKAP-1 knock-down induced by pressure overload induces mitochondrial dysfunction, increases ROS levels and promotes cardiomyocyte death^17^ as well as mitochondrial fragmentation^21^. However, the possible beneficial role of AKAP-12 remains controversial. AKAP-12 is known as a tumor suppressor protein^22^ that exerts protective effects in fibrotic scars during central nervous system repair^23^ and inhibits the activity of hypoxia inducible factor-1α^24^. Moreover, deficiency of AKAP-12 causes hemorrhage in embryos of zebrafish^25^ and increases the susceptibility to injury-induced glomerulonephritis^26^. Nevertheless, AKAP-12 is specifically induced on exposure of endothelial cells to hypoxia^27^ and down-regulation of AKAP-12 activates the antioxidant thioredoxin-1 expression and improves angiogenic gene expression^28^. Our results show that AKAP-12 was down-regulated by Aldo in cardiac fibroblasts *in vitro* and *in vivo* as well as in myocardial biopsies from pressure overloaded myocardium (AS). Moreover, Aldo circulating levels negatively correlated to myocardial AKAP-12 expression in myocardial biopsies, reinforcing that Aldo could be one of the negative regulators of AKAP-12 in myocardium. Interestingly, AKAP-12 over-expression blunted Aldo effects on mitochondrial function and oxidative stress parameters in cardiac cells, reinforcing the hypothesis that the down-regulation of AKAP-12 leads to mitochondrial dysfunction and oxidative stress induction in human cardiac fibroblasts. AKAP-12 down-regulation triggered by Aldo may represent an important event in the development of cardiac alterations. Thus, the identification of molecules that restore AKAP-12 levels in myocardium is expected to be beneficial.

Of special interest, Aldo and AKAP-12 knock-down were both able to decrease PGC-1α expression as well as mtDNA in human cardiac fibroblasts. In line with our observations, Aldo time-dependently decreases PGC-1α and mtDNA in human proximal tubular cells, inducing mitochondrial dysfunction^14^. In our study, myocardial PGC-1α expression was lower in Aldo-treated rats and in AS patients, and cardiac AKAP-12 associated to PGC-1α. Accordingly, it has been described that pressure overload (condition mimicking AS) decreases cardiac PGC-1α expression^29^, and PGC-1α KO mice subjected to pressure overloads presents increased oxidative stress and a decline in mitochondrial function^30^. Interestingly, in AS patients, PGC-1α mRNA levels in myocardial biopsies directly correlate with PGC-1α expression measured in peripheral blood^31^. AS patients with low PGC-1α present an inflammatory profile whereas AS patients with high PGC-1α exhibit better antioxidant status^31^. Consequently, reduced PGC-1α expression levels found in myocardial biopsies from AS patients correlated with decreased mitochondrial function. Thus, the fact that Aldo and AKAP-12 decreases PGC-1α expression may play a significant role in the development of mitochondrial dysfunction.

In summary, the present study shows that Aldo-induced oxidative stress could be mediated by AKAP-12 down-regulation that leads to PGC-1α reduction and to mitochondrial dysfunction.

## Methods

### Cell culture

Adult human cardiac fibroblasts were obtained from Promocell, used between passages 5–7 and cultured according to the manufacter’s instructions. Cells were stimulated with Aldo (10^−8^ M, Sigma) for 24 h using Mass spectrometry-based quantitative proteomics as previously reported^19,32^.

### Clustered Regularly Interspaced Short Palindrome Repeats/Cas9 Genome Editing- Mediated Deletion of AKAP-12

The knockdown/activation of AKAP12 in cells was performed by clustered regularly interspaced short palindrome repeats (CRISPR)/Cas9-guided genome editing as previously reported^19,32^.

### Western blot analysis

Proteins were electrophoresed on SDS polyacrylamide gels, transferred to Hybond-c Extra nitrocellulose membranes (Bio-Rad) and incubated with primary antibodies for: AKAP-12 (Santa Cruz Biotechnology; 1:100), Nitrotyrosine (Santa Cruz Biotechnology; 1:100), CML (Abcam; 1:200), MDA (Abcam; 1:200), PGC1-α (Santa Cruz Biotechnology; 1:100), PHB (Cell Signaling; 1:100). After washing, detection was made through incubation with peroxidase-conjugated secondary antibody, and developed using an ECL chemiluminescence kit (Bio-Rad). Results are expressed as an n-fold increase over the values of the control group in densitometric arbitrary units. All Western Blots were performed at least in triplicate for each experimental condition.

### Real-time reverse transcription PCR

Total RNA was extracted with Trizol Reagent (Euromedex) and purified using the RNeasy kit, according to the manufacturer’s instructions (Qiagen). Quantitative PCR analysis was then performed with SYBR green PCR technology (Bio-Rad) (Supplemental Table 1). Data were normalized by HPRT and β-actin levels or nuclear DNA and expressed as percentage relative to controls. All PCRs were performed at least in triplicate for each experimental condition.

### *In vivo* studies

Adult male Wistar rats were treated for 3 weeks with vehicle (n = 10), Aldo-salt (Sigma, 1 mg/kg per day and 1% NaCl as drinking water; n = 10), Aldo-salt + Spironolactone (Sigma, 200 mg/kg per day; n = 10)^33,34^.

The Animal Care and Use Committee of Universidad Complutense de Madrid and Dirección General de Medio Ambiente, Comunidad de Madrid (PROEX 242/15) approved all experimental procedures according to the Spanish Policy for Animal Protection RD53/2013, which meets the European Union Directive 2010/63/UE.

### Patient population

Serum samples and myocardial biopsies were obtained from AS patients (n = 26), referred to our center for aortic valve replacement. All patients were evaluated by echocardiography. As controls, myocardial biopsies from subjects who have died from non-cardiovascular-related diseases were obtained at autopsy (Control, n = 13)^35^. Informed consent was obtained from each patient and control and the study protocol conforms to the ethical guidelines of the 1975 Declaration of Helsinki as reflected in a priori approval by the institution’s human research committee (Comité ético de experimentación clínica. Gobierno de Navarra. Departamento de Salud; Pyto2015/26).

### ELISA

Total antioxidant capacity and peroxide production were measured following manufacturer’s instructions (Sigma Aldrich). The results were normalized to the control condition. Data were expressed as a fold change relative to control conditions for *in vitro* and animal studies.

### Immunohistological evaluation

Histological determinations in cardiac tissue were performed in 5 μm-thick sections. Slides were treated with H_2_O_2_ and incubated with AKAP-12 (Santa Cruz; 1:100), Nitrotyrosine (Santa Cruz; 1:5000), CML (Abcam; 1:2000), PGC-1α (Santa Cruz; 1:100) washed three times, and then incubated with the horseradish peroxidase-labeled polymer conjugated to secondary antibodies (Dako Cytomation). The signal was revealed by using DAB Substrate Kit (BD Pharmingen). As negative controls, samples followed the same procedure described above but in the absence of primary antibodies. All measurements and quantifications were performed blind in an automated image analysis system (Nikon).

### Statistical analyses

Data are expressed as mean ± SEM. Normality of distributions was verified by means of the Kolmogorov-Smirnov test. The unpaired Student’s t test or the Mann Whitney U tests were used to assess statistical differences between two experimental conditions. The predetermined significance level was P < 0.05.

Spearman’s correlation coefficients were calculated to determine correlations. The predetermined significance level was P < 0.05.

## Electronic supplementary material

Supplemental material
