# Supplementary material for: Aldosterone Impairs Mitochondrial Function in Human Cardiac Fibroblasts via A-Kinase Anchor Protein 12
Source: Sci Rep. 2018 May 1;8:6801. doi: 10.1038/s41598-018-25068-6 (PMC5931570; doi:10.1038/s41598-018-25068-6)
Supplement: Supplementary file 1 — Supplemental material [file 41598_2018_25068_MOESM1_ESM.doc]

**ALDOSTERONE IMPAIRS MITOCHONDRIAL FUNCTION IN HUMAN CARDIAC FIBROBLASTS VIA A-KINASE ANCHOR PROTEIN 12**

Jaime Ibarrola, Rafael Sádaba, Ernesto Martinez-Martinez, Amaia Garcia-Peña, Vanessa Arrieta, Virginia Alvarez, Amaya Fernández-Celis, Alicia Gainza, Victoria Cachofeiro, Enrique Santamaría, Joaquin Fernández-Irigoyen, Frederic Jaisser, Natalia López-Andrés

**Supplemental table**

**Table S1: Primers used for real time PCR analysis**

| **Gene** | **Primer** | **Sequence (5´to 3´)** |
| --- | --- | --- |
| **AKAP-12** | Forward | AGC GTC GGG TCT GAA GAA AG |
|  | Reverse | AAG CTT TCT GGG GTG GTC TG |
| **PGC-1α** | Forward | TCC CGA TCA CCA TAT TCC |
|  | Reverse | TTC AAG AGC AGC AAA AGC |
| **PHB** | Forward | GCA GGA CAT TGT GGT AGG GG |
|  | Reverse | GCT GGT GAA GAT GCG AGG AA |
| **Total mtDNA** | Forward | CAC CCA AGA ACA GGG TTT GT |
|  | Reverse | TGG CCA TGG GTA TGT TGT TA |
| **nucDNA** | Forward | TGC TGT CTC CAT GTT TGA TGT ATC T |
|  | Reverse | TCT CTG CTC CCC ACC TCT AAG T |
| **HPRT** | Forward | TTG CTT TCC TTG GTC AGG CA |
|  | Reverse | ATC CAA CAC TTC GTG GGG TC |
| **β-actin** | Forward | GCC GCC AGC TCA CCA T |
|  | Reverse | TCG ATG GGG TAC TTC AGG GT |
